# Morphological, physicochemical, and antioxidant profile of noncommercial banana cultivars

**DOI:** 10.1002/fsn3.208

**Published:** 2015-02-17

**Authors:** Tonna A Anyasi, Afam IO Jideani, Godwin A Mchau

**Affiliations:** 1 Department of Food Science and Technology, School of Agriculture, University of VendaPrivate Bag X5050, Thohoyandou, 0950, Limpopo Province, South Africa; 2 Department of Horticultural Sciences, School of Agriculture, University of VendaPrivate Bag X5050, Thohoyandou, 0950, Limpopo Province, South Africa

**Keywords:** Antioxidant activity, banana cultivars, characterization, morphology, titratable acidity, total phenolic content

## Abstract

Banana cultivars––Luvhele (*Musa*ABB), Mabonde (*Musa*AAA), and Muomva-red (*Musa balbisiana*) ––were characterized for morphological, physicochemical, and antioxidant properties. All three cultivars varied significantly (*P *<* *0.05) in their morphology, pH, titratable acidity and total soluble solids with no significant difference in their ash content. Individual cultivars showed variations in flour starch granule when observed using a scanning electron microscope. Characterization of cultivars for total polyphenols (TPs) and antioxidant activity upon pretreatment with ascorbic, citric, and lactic acid shows that the 1,1-diphenyl-2-picrylhydrazyl (DPPH) radical scavenging assay of samples varied significantly as Muomva-red cultivar (1.02 ± 0.01 mg GA/g) expressed the highest DPPH activity at lactic acid concentration of 20 g/L. Total polyphenol content was also highest for Muomva-red [1091.76 ± 122.81 mg GAE/100 g (d.w.)]. The high amount of TPs present in these cultivars make them suitable source of bio-nutrients with great medicinal and health functions.

## Introduction

Banana is an edible fruit grown in tropical and subtropical regions of the world at latitude 20° close to the equator, with characteristic seasonal variation in rainfall and temperature (Pua 2007). Globally, there exist more than a 100 common names for which the fruit from the genus *Musa* is associated, with over 1000 cultivars and landraces emanating from more than fifty *Musa* species (Heslop-Harrison and Schwarzacher 2007; Arvanitoyannis and Mavromatis 2009). The name banana is said to originate from the coastal part of West Africa, Guinea or Sierra Leone, and was accepted in the New World as a term used for describing the fruit's peel. The oldest record of edible banana has been traced to originate from India (600 B.C.). Banana, apart from apple, is the highest consumed fruit in Europe (Arvanitoyannis and Mavromatis 2009) and it can either be classified as commercial or noncommercial cultivars.

**Figure 1:**
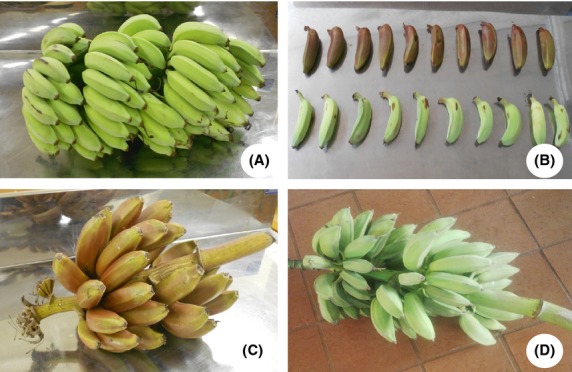
Some noncommercial banana cultivars in Limpopo Province of South Africa: (A) Luvhele banana bunch; (B) Muomva-red and Luvhele banana fingers; (C) Muomva-red banana bunch; and (D) Mabonde banana bunch.

The noncommercial cultivars also referred to as indigenous varieties are so-called due to the fact that they are rarely cultivated for export or trade, but are grown in household gardens by small-scale growers mostly for consumption (Anyasi et al. 2013). Basically, two major noncommercial cultivars are grown in Limpopo Province of South Africa: Mabonde and Luvhele cultivars (Fig.1). Muomva-red, another noncommercial variety cultivated in Limpopo Province, is also grown in other parts of the world. Variations exist among these noncommercial varieties in their morphological features of length, width, peel and pulp colour, weight, and overall shape as well as their antioxidant properties. These properties are factors that are used in the classification of these fruits to their corresponding groups and subgroups. Commercial cultivars are known to be larger in size, weight, length, and overall shape and have higher consumer acceptability when compared to the noncommercial cultivars. Although the fruit's final shape and size are representatives of the cultivars, they are also affected by environmental and genetic interactions (Robinson and Sauco 2010). Accordingly, the commercial varieties have been grouped into various cultivars using these parameters due majorly to their acceptability as well as nutritional content. However, the noncommercial cultivars are rarely known or classified even though different cultivars exist.

Current trends show an increase in research on the utilization of unripe banana products for use by consumers due to the presence of polyphenols (Aurore et al. 2009), free and bound phenols such as anthocyanins in fruit pulp (Bennett et al. 2010), and moderate antioxidant capacity in flour of unripe green banana (Menezes et al. 2011; Sarawong et al. 2014). Nutritional differences have also been observed in the mineral and bioactive profiles of these fruits. Literatures studied show that some of these noncommercial cultivars contain higher antioxidant nutrients when compared to the commercial cultivars (Faller and Fialho 2010; Fu et al. 2011). However, there is scarce information on variation in morphological, physicochemical, and antioxidant properties that exist among these noncommercial banana varieties cultivated in South Africa and other tropical and subtropical countries. This research therefore seeks to comparatively profile the morphological, physicochemical, and antioxidant properties of three noncommercial banana cultivars.

## Materials and Methods

### Plant materials and treatments

A total of 30 banana fingers, each selected randomly from different parts of six fruit bunches of three noncommercial banana cultivars: Luvhele, Mabonde and Muomva-red and obtained at the unripe green stage 2 of ripeness (Aurore et al. 2009) from household banana farms in Thulamela Municipality, Vhembe District of South Africa, were used for this research. The fingers were randomly collected from two bunches of each of the individual banana fruits. Characterization of all three cultivars was done by determining the morphological parameters of finger length and girth, physicochemical properties, total polyphenol, as well as antioxidant properties of the fruits. Noncommercial cultivars were also compared with information from *Musa* Germplasm Information System (MGIS) as well as from other literatures (Daniells et al. 2001; Aurore et al. 2009; Anyasi et al. 2013). The cultivar names, their species, subspecies, accession names, and numbers obtained from the MGIS database and other literatures are shown in Table1.

**Table 1:** Identification of noncommercial banana cultivars studied

Banana cultivar	MGIS documentation			
	Species	Subspecies	Accession name	Accession number
Luvhele	ABB	Pisang Awak	Ducasse	SJR0009
Mabonde	AAA	Lujugira	Entente	KAW0001
Muomva-red	*Balbisiana*	–	Pisang Klutuk Wulung	NYO0033

Source: Daniells et al. (2001); Aurore et al. (2009); Anyasi et al. (2013).

To obtain flour used for the determination of phenolic and antioxidant properties, pulp of noncommercial cultivars was cut to 4 mm size and pretreated with organic acids; ascorbic, citric, and lactic acid at concentrations of 10, 15, and 20 g/L for 10 min. The mixture containing the pretreatment and sliced pulp was allowed to drain for 2 min. Fruit pulp was then conventionally dried in an air oven dryer (Prolab instruments, South Africa) at a temperature of 70°C for 12 h. Dried pulp was later milled (Retsch ZM 200 miller, Haan, Germany) at 16,000 rpm for 30 sec. Banana flour obtained from milled pulp was then used to determine the total polyphenol and antioxidant activity of banana cultivars.

### Determination of fruit length and girth

Fruit length and girth were determined from a selected banana bunch using the protocols of Dadzie and Orchard (1997). From two bunches of noncommercial banana cultivars, 10 randomly selected individual fingers from different hands, top to bottom of banana bunch, were used to determine fruit length and girth. Fruit length determination involves measuring the outer and inner curve of individual fruits with a tape from the distal end of the fruit to the point at the proximal end where the fruit pulp is judged to terminate. Fruit girth: distal end, widest midpoint, and proximal end were determined by measuring individual fruit with a tape at the widest midpoint, distal, and proximal end of each individual fruit finger (Dadzie and Orchard 1997).

### Total soluble solids and titratable acidity

The total soluble solids (TSS) of fruit cultivars were determined using the methods of Dadzie and Orchard (1997). Approximately 30 g of banana pulp tissue was homogenized in 90 mL of distilled water for 2 min and filtered using Whatman No.1 filter paper. A single drop of the filtrate was placed on the prism of a refractometer and readings for percentage TSS were taken. Recorded values were multiplied by three due to the dilution factor of the pulp, which is three times the amount of distilled water. For the determination of total titratable acidity (TTA), from 100 mL of filtered banana pulp, 10 mL was pipette into a conical flask, and diluted to about 80 mL with distilled water. About 0.3 mL phenolphthalein was then added to the solution titrated to a faint pink end-point with 0.1 N NaOH. TTA was expressed as percentage malic acid.

### pH and ash content

The pH was determined using the protocols of Dadzie and Orchard (1997). Approximately 30 g of banana pulp tissue was homogenized with 90 mL of distilled water for 2 min and filtered using Whatman No 1 filter paper. Readings were recorded by inserting the pH electrode in the filtrate on stabilization of sample filtrate. Ash content of fruit sample was determined using method of Horwitz (2000). Empty crucibles were placed in a muffle furnace at 600°C for an hour, cooled in desiccators, and weighed. About 1 g of each sample of banana flour was placed in the crucible and ignited over a burner with the help of a blowpipe until it was charred. The principle was placed in a muffle furnace at 600°C for 2–4 h. The appearance of gray white ash indicates complete oxidation of all organic matter in the sample.

### Scanning electron microscopy (SEM)

Imaging of unripe fruit flour was conducted using a Leo 1430VP SEM. Prior to imaging, flour samples were mounted on a stub with double-sided carbon tape. Samples were then coated with a thin layer of gold in order to make flour surface electrically conducting. SEM micrographs revealed the surface structure of banana flour of all three cultivars. Beam conditions during surface analysis were 7 kV and approximately 1.5 nA, with a spot size of 150 and a magnification of 1000 kX.

### Total polyphenol

Total polyphenols of flour obtained from the three noncommercial banana cultivars was determined using the Folin-Ciocalteu colorimetric methods of Prabhu and Barrett (2009) with slight modifications. The method is based on the reduction of MoO^4+^ to MoO^3+^ that is detected by color change from yellow to blue; measured at 760 nm. Approximately 0.2 g of milled oven dried fruit pulp was weighed and 2 mL of acetone was added. The mixture was incubated for 1 h at room temperature, shaking occasionally and centrifuged at 6000 rpm for 5 min at 4°C. To 9 *μ*L of centrifuged sample in a microplate, 109 *μ*L of Folin-Ciocalteu solution was added. About 180 *μ*L of 7.5% Na_2_CO_3_ was added to the mixture, covered with aluminium foil and incubated at 50°C for 5 min. Absorbance was read at 760 nm, using an UV spectrophotometer microplate reader (Zenyth 200rt Biochrom, UK). Gallic acid was used as the standard phenol compound and acetone used as the extraction solvent. The results were expressed as equivalents of gallic acid (mg GAE/100 g d.w.) from the calibration curve.

### Determination of 1,1-diphenyl-2-picrylhydrazyl (DPPH) scavenging activity

The ability of the banana flour to scavenge the unstable free radical 1,1-diphenyl-2-picrylhydrazyl was determined using the methods of Ribeiro et al. (2008). This capacity to scavenge the stable DPPH free radical can be used in expressing the measure of antioxidant activity in fruits (Musa et al. 2013). Methanol was used as the extraction solvent and gallic acid used as standard with result of analysis measured in mg GA/g (d.w.). To 0.2 g of milled banana flour, 2 mL of methanol was added to the sample, incubated for 30 min at room temperature and centrifuged at 6000 rpm for 10 min at 4°C. Dilution of different concentrations of 10, 20, 30, 40, and 50 mg/mL of the sample was used to determine the IC_50_ of the sample. Final values of IC_50_ were obtained by plotting the percentage disappearance of DPPH as a function of the sample concentration. To 250 *μ*L of DPPH solution, 28 *μ*L of sample mixture was added in a microplate, covered with aluminum foil and incubated for 1 h at room temperature. Absorbance was read at 517 nm using an UV spectrophotometer microplate reader (Zenyth 200rt Biochrom, UK).

### Statistical Analysis

All measurements were conducted in triplicate and results presented as mean values ± standard deviation (SD). Statistical analysis was conducted using the one-way analysis of variance (ANOVA) and means of results for each experiment was compared using the Tukey Honest Significant Difference (HSD) Test (*P < *0.05 confidence levels). SPSS 21 for windows (SPSS Inc., Chicago, IL) statistical software package was used to conduct the statistical analysis.

## Results and Discussion

### Fruit morphology

**Figure 2:**
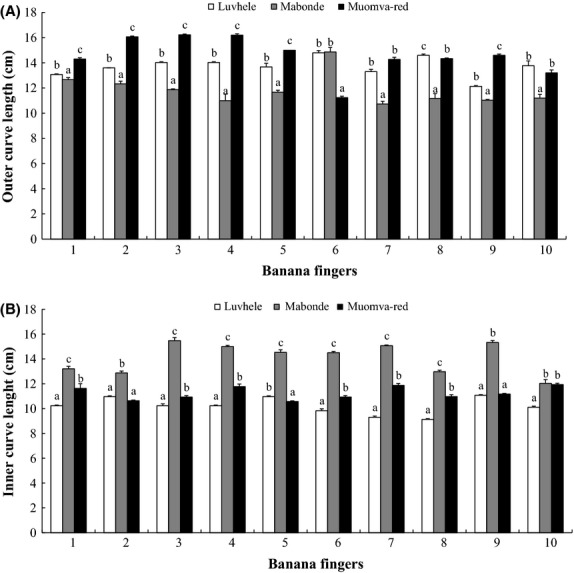
(A) Outer curve length of individual banana fingers; (B) Inner curve length of individual banana fingers. Data labels with different letters are significantly different (*P *<* *0.05) for individual fruit fingers using Tukey HSD test. Error bars are standard deviation of means (*n* = 3).

Differences that exist in fruit morphology are due mostly to variations in cultivars. Banana cultivars differ in their fruit girth measurements with different cultivars showing different sizes and shapes in those parts of the banana peel (Fig.2A). The outer curve length of Mabonde had the lowest length in almost all fingers when compared statistically (*P *<* *0.05) to Luvhele and Muomva-red cultivars. Muomva-red banana outer curve length was highest for fingers 1, 2, 3, 4, 5, 7, and 9 when compared to other cultivars and differed significantly (*P *<* *0.05) from Luvhele and Mabonde. The outer curve length of Luvhele was significantly the smallest except for finger 6 when compared to other cultivars. Cultivar Muomva-red is thus said to have significantly longer banana fingers when compared to the other noncommercial cultivars used in this study. Results of finger length of individual commercial banana fingers show that the commercial variety such as Williams and Grand Nain were longer in length: 17–24 cm (Daniells et al. 2001) when compared to the noncommercial cultivars used in this study.

Results from the inner curve length showed that Mabonde banana had the highest inner curve length with finger 3 (15.47 ± 0.25 cm) and varied significantly (*P *<* *0.05) from other noncommercial cultivars. There was a marked significant difference in the inner curve length of Mabonde banana cultivar in all fingers of the three cultivars. The inner curve length of both Luvhele and Muomva-red were significantly different (*P *<* *0.05) for all fingers except for fingers 2 and 9 (Fig.2B). Similar results were reported by Belayneh et al. (2014) on the fruit length of some noncommercial cooking bananas.

The distal end, widest midpoint, and proximal end of banana fruits are parameters that make up the girth and morphological structure of the fruit. These morphological properties could be used for characterization and differentiation of one banana cultivar from another (Dadzie and Orchard 1997). Among all three cultivars analyzed, there were significant differences in banana distal end with Mabonde and Muomva-red varying significantly in very few fingers. Mabonde banana cultivar had the widest midpoint for all cultivars, varying significantly (*P *<* *0.05) from other cultivars (Table2). The widest midpoint of Muomva-red was significantly higher than that of Luvhele for all fingers measured. The proximal end varied significantly in all three cultivars apart from finger 1 in which there was no significant difference in the measured proximal end. Results obtained on fruit girth for all cultivars agree with results obtained by Belayneh et al. (2014) on the fruit girth of some cooking bananas.

**Table 2:** Fruit girth of noncommercial banana cultivars

Banana fingers	Distal end (cm)			Widest midpoint (cm)			Proximal end (cm)		
	Luvhele	Mabonde	Muomva-red	Luvhele	Mabonde	Muomva-red	Luvhele	Mabonde	Muomva-red
1	4.70 ± 0.36^ab^	4.60 ± 0.17^a^	5.13 ± 0.06^ab^	10.27 ± 0.06^i^	12.67 ± 0.06^k^	11.50 ± 0.00^j^	4.07 ± 0.15^x^	4.10 ± 0.10^x^	4.23 ± 0.06^x^
2	4.13 ± 0.12^a^	4.77 ± 0.25^b^	4.87 ± 0.38^b^	10.33 ± 0.06^i^	12.77 ± 0.06^k^	11.23 ± 0.06^j^	4.27 ± 0.06^x^	4.33 ± 0.15^x^	4.20 ± 0.10^x^
3	4.27 ± 0.06^a^	4.90 ± 0.44^a^	5.07 ± 0.47^a^	10.33 ± 0.06^i^	13.57 ± 0.06^k^	11.90 ± 0.00^j^	4.60 ± 0.10^y^	4.57 ± 0.35^y^	3.77 ± 0.15^x^
4	4.23 ± 0.06^a^	4.50 ± 0.00^a^	5.47 ± 0.32^b^	10.10 ± 0.00^i^	14.00 ± 0.1^k^	12.30 ± 0.00^j^	4.47 ± 0.06^xy^	4.73 ± 0.25^y^	4.20 ± 0.10^x^
5	4.17 ± 0.06^a^	4.73 ± 0.15^b^	4.23 ± 0.12^a^	10.27 ± 0.06^i^	15.60 ± 0.00^k^	11.43 ± 0.06^j^	4.67 ± 0.06^y^	5.83 ± 0.21^z^	3.83 ± 0.12^x^
6	3.80 ± 0.00^a^	4.67 ± 0.15^b^	5.10 ± 0.17^b^	10.27 ± 0.00^i^	14.23 ± 0.06^k^	11.83 ± 0.12^j^	4.50 ± 0.06^x^	5.47 ± 0.25^y^	4.27 ± 0.23^x^
7	3.97 ± 0.06^a^	5.17 ± 0.32^b^	5.13 ± 0.32^b^	10.20 ± 0.00^i^	13.90 ± 0.00^k^	11.60 ± 0.00^j^	4.60 ± 0.10^y^	5.17 ± 0.15^z^	4.10 ± 0.10^x^
8	3.70 ± 0.10^a^	4.83 ± 0.25^bc^	4.57 ± 0.21^b^	10.20 ± 0.10^i^	13.53 ± 0.12^k^	12.17 ± 0.06^j^	4.50 ± 0.10^y^	5.37 ± 0.06^z^	4.00 ± 0.20^x^
9	3.87 ± 0.06^a^	4.53 ± 0.21^b^	3.87 ± 0.15^a^	9.93 ± 0.06^i^	13.23 ± 0.42^k^	11.90 ± 0.10^j^	4.07 ± 0.06^y^	3.93 ± 0.21^xy^	3.73 ± 0.06^x^
10	3.83 ± 0.06^a^	4.63 ± 0.15^b^	4.53 ± 0.15^b^	10.17 ± 0.12^i^	12.17 ± 0.06^k^	11.50 ± 0.10^j^	4.27 ± 0.06^x^	3.90 ± 0.10^y^	4.10 ± 0.10^xy^

Means with different superscript across rows for each fruit girth are significantly different (*P *<* *0.05) using the Tukey HSD test. Values are means ± standard deviation (*n* = 3).

### Physicochemical properties of fruit cultivars

The total titratable acidity (TTA) of cultivars analyzed showed that there was no significant difference in the TTA of Luvhele (1.61 ± 0.13) and Muomva-red (1.65 ± 0.15). Cultivar Mabonde showed marked significant difference in its TTA when compared to other noncommercial cultivars (Table3). Similar results for TTA were reported by Belayneh et al. (2014), while lower TTA values were recorded for unripe Robusta banana cultivar after 6 days of storage upon pretreatment of the individual banana fingers with ethrel solution (Kulkarni et al. 2011). According to Sadler and Murphy (2010), TTA is used in the determination of total acid concentration present in food products. In fruits, acidity decreases with concomitant rise in maturity, thus TTA and sugar content of fruits acts as an important parameter in the determination of both flavour and overall maturity of the fruit.

**Table 3:** Physicochemical properties of unripe noncommercial banana cultivars

Properties (f.w.)	Banana cultivars		
	Luvhele	Mabonde	Muomva-red
pH	6.12 ± 0.03^c^	5.46 ± 0.02^b^	5.36 ± 0.02^a^
Total titratable acidity	1.61 ± 0.13^a^	3.40 ± 0.08^b^	1.65 ± 0.15^a^
Total soluble solids (°Brix)	1.77 ± 0.03^b^	1.49 ± 0.05^a^	2.92 ± 0.14^c^
Ash (%)	1.13 ± 0.05^a^	1.22 ± 0.58^a^	1.33 ± 0.11^a^

Means with same superscript across rows are not significantly different (*P *<* *0.05) using Tukey HSD test. Values are means ± standard deviation (*n* = 3).

Total soluble solids (TSS) varied significantly (*P *<* *0.05) in all noncommercial banana cultivars. According to Dadzie and Orchard (1997), TSS can be used in the screening of different banana hybrids. Generally, the amount of TSS in a fruit is directly proportional to the degree of fruit ripeness as TSS is said to increase with fruit ripeness. Thus, TSS can also serve as a useful index in the determination of fruit maturity and ripeness. Cultivar red exhibited a significantly higher TSS (2.92 ± 0.14) when compared to other fruit cultivars. Cultivar Mabonde had the least soluble sugar content (1.49 ± 0.05) when compared with all three cultivars. Similar results were recorded for TSS of green Cavendish banana flour (Alkarkhi et al. 2011) and unripe banana (Kulkarni et al. 2011). The low values recorded for the TSS of all cultivars are attributed to the fact that the available starch present in the unripe cultivars are yet to be converted into soluble sugars through enzymatic degradation (Zhang et al. 2005). Degradation and consequent reduction in starch proceed rapidly during the onset of ripening thus leading to an overall increase in TSS of fruits.

pH of all analyzed samples varied significantly (*P *<* *0.05) in all fruit cultivars. Cultivar Luvhele was the least acidic in all cultivars (6.12 ± 0.03) when compared to Mabonde (5.46 ± 0.02) and Muomva-red (5.36 ± 0.02) on fresh weight basis. Muomva-red was more acidic when compared to the other cultivars, which agrees with values obtained by Kulkarni et al. (2011) for unripe banana cultivars. Alkarkhi et al. (2011) also report similar results for the pH of unripe green banana flour. Conversely, the pH values of all unripe cultivars were higher than values obtained in ripe cultivars as reported by Arvanitoyannis and Mavromatis (2009) due to the associated increase in organic acids present in fruits as ripening increases. The pH of food measures the amount of hydronium ions (H_3_O^+^) present in a food produce. Many food quality criteria have been found to correlate better with pH than with acid concentration (Sadler and Murphy 2010).

Cultivar Luvhele had a percentage ash content of 1.13 ± 0.05, cultivar Mabonde 1.22 ± 0.58 and Red 1.33 ± 0.11 (f.w.). The result also agrees with that of Nwokocha and Williams (2009) on white and yellow plantain. Results also showed that there was no significant difference (*P *<* *0.05) in the percentage ash of all unripe banana cultivars analyzed. Ash content in fruits and vegetables are affected by agro-climatic conditions such as cultivation practices, nature of soil, and climatic conditions. Ash content is used to determine the total mineral present in a food produce. A high percentage ash value equals a high total mineral value in the fruit sample. Mineral availability in fruits and vegetables are influenced positively or negatively by these agro-climatic conditions (Forster et al. 2002). The absence of marked differences in the nature of soil and climatic conditions are factors that explain the lack of difference in the ash content of all cultivars examined (Bugaud et al. 2006). All three banana cultivars were obtained at the same location with the same soil and climatic conditions applied during cultivation.

### SEM analysis of fruit flour

**Figure 3:**
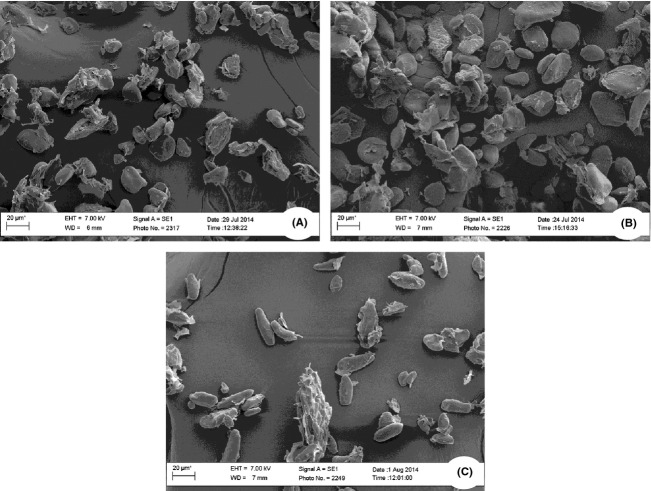
SEM micrographs of flour showing starch granules in citric acid pretreated unripe banana cultivars. (A) Luvhele; (B) Mabonde; and (C) Muomva-Red. Magnification = 1000 kX.

Surface morphology obtained from SEM micrographs of all three cultivars showed marked variations in structure, shape, and size of starch granules of the different banana flour. Electron micrographs suggest that granules were considerably irregular in their structures among cultivars. Observed shapes include polygonal for Luvhele, oval for Mabonde and elongated for Muomva-red unripe banana flour (Fig.3). Oval shape of granules obtained from unripe Mabonde flour is in agreement with the work of Utrilla-Coello et al. (2014) which showed starch granules exhibiting regular shapes with oval appearance. Drying temperature of 70°C for 12 h during flour production, as well as differences in cultivars (Sivak and Preiss 1998; Jackson 2003) account for the variations in granule morphology among the different cultivars. Irregular shapes and size of granules as observed in micrographs could be attributed to high heat treatment leading to irreversible swelling, puncturing, and gelatinization of banana flour.

**Figure 4:**
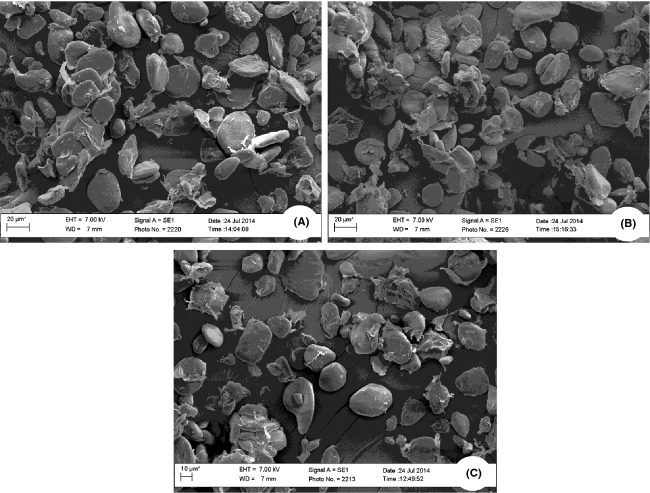
SEM micrographs of flour showing starch granules in unripe Mabonde cultivar pretreated using GRAS organic acids. (A) Ascorbic acid pretreated; (B) Citric acid pretreated; and (C) Lactic acid pretreated. Magnification = 1000 kX.

Pretreatment with GRAS organic acids of individual cultivars, however, showed no difference in shape and structure of granules obtained from flour as all cultivars retained their shape and size (Fig.4). Adhesion between granules was also observed in flour samples of all cultivars irrespective of pretreatment concentration. This result is in agreement with Wang and Copeland (2012) on effect of alkali treatment on structure and function of pea starch granules. Presence of adhesion between granules can be attributed to the occurrence of lipid and protein molecules in the granules of unripe flour samples (Perez et al. 2009).

### Total phenolics of unripe banana cultivars

**Figure 5:**
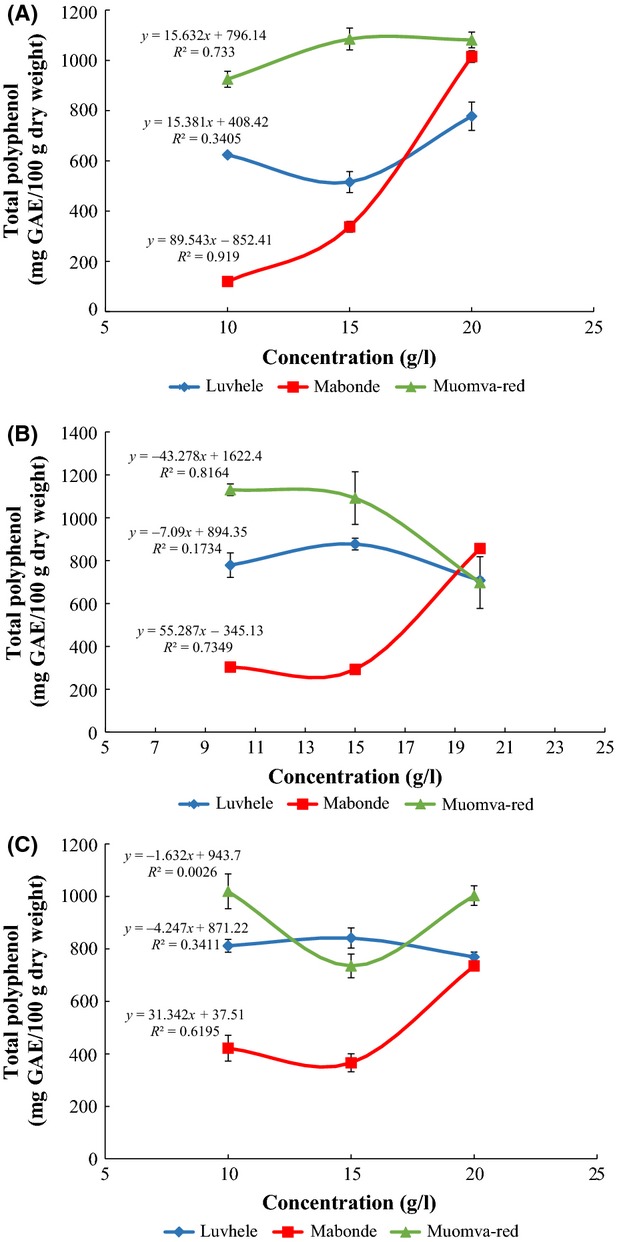
(A) Relationship between total polyphenol content (TPC) and ascorbic acid pretreatment of noncommercial banana cultivars; (B) Relationship between TPC and citric acid pretreatment of noncommercial banana cultivars; and (C) Relationship between TPC and lactic acid pretreatment of noncommercial banana cultivars. Error bars are standard deviation of means (*n* = 3).

Fruit samples with different pretreatment showed various total polyphenol content (TPC) at varying levels of pretreatment concentration. Cultivar Mabonde had a high coefficient of determination (*r*^2^ = 0.919) for all organic acid treatment with ascorbic acid treatment recording the highest relationship between treatment and cultivars (Fig.5A) for fruit TPC. Weak relationship (*r*^2^ = 0.1734) was observed between cultivar Luvhele and citric acid treatment (Fig.5B) and very weak relationship (*r*^2^ = 0.0026) between cultivar Muomva-red with lactic acid treatment (Fig.5C). There was significant increase in TPC yield in all cultivars as the concentration of pretreatment increased. Cultivar Muomva-red showed the highest amount of TPC in all pretreatment and at different levels of concentration except for concentration levels of citric acid 20 g/L and lactic acid 15 g/L (Fig.5B and C). Cultivar Luvhele showed a significantly higher TPC yield of 707.87 ± 12.62 mg GAE/100 g and 841.59 ± 38.39 mg GAE/100 g at such concentration levels. Also fruits treated with citric acid had a higher TPC yield in all cultivars with pretreatment concentration of 15 mg/L having the highest significant yield (*P *<* *0.05) of TPC for Muomva-red. Generally, there were significant differences in yield of TPC in all cultivars at all treatments except for pretreatment concentration of citric acid 20 g/L where there was no significant difference in TPC across all cultivars. Results also show that TPC was highest for Muomva-red with a value of 1091.76 ± 122.81 mg GAE/100 g (d.w.). Significant variations that exist in quantity and quality of total polyphenols in plant foods have been attributed to diverse inherent and external conditions such as genetic composition, plant cultivar, soil composition, state of plant maturity, and postharvest practices (Jaffery et al. 2003; Faller and Fialho 2010).

The TPC values obtained from the noncommercial cultivars were higher than those obtained by Sarawong et al. (2014) with TPC of native banana flour at 220.30 ± 0.59 mg GAE/100 g (db) and Fu et al. (2011) who reported values of 57.13 ± 3.64 mg GAE/100 g (general banana), 29.07 ± 2.06 mg GAE/100 g (cooking banana), and 25.55 ± 0.60 mg GAE/100 g (royal banana). Similarly, Menezes et al. (2011) showed lower total polyphenols of 50.65 ± 0.80 mg GAE/100 g (d.w.) in flour of unripe banana. Anusuya et al. (2013) also recorded polyphenol content (110.45 mg GAE/100 g, 94.03 and 79.92 mg GAE/100 g) from banana pseudo stem flour when compared to results obtained from this present study. The high TPC content in the noncommercial banana cultivars, especially Muomva-red could be attributed to the colour of the banana cultivars which has a red peel when unripe. This could also be seen in almost all the pretreatment concentration where Muomva-red significantly exhibited the highest amount of TPC.

High temperature processing leads to alteration of the molecular compounds resulting in polymerization and alteration of the molecular structure of phenolic compounds thus leading to a reduced extractability (Altan et al. 2009; Brennan et al. 2011; Nayak et al. 2011; Sharma et al. 2012). The high phenolic contents in all fruit cultivars analyzed agree with Sarawong et al. (2014) who argue that increase in phenolics could be due to the disruption of cell walls by all extrusion conditions thus resulting in higher TPC content. Accordingly, the TPC of fruits and plant produce is generally dependent on part of the plant and the solvent used for extraction. Apart from varietal difference, high TPC yield in all cultivars could also be as a result of pretreatment with organic acid used in the preparation of unripe banana flour. Sulaiman et al. (2011) stated that significant differences observed in the phenolic content among different cultivars can be attributed to the breakdown of phenols influenced by varietal differences.

### Antioxidant properties of unripe banana cultivars

Antioxidant capacity of all noncommercial banana cultivars was determined by DPPH assay. Results of analysis showed that the antioxidant capacity varied significantly in all cultivars and at different organic acid pretreatment concentration (Table4). Mabonde cultivar recorded the lowest DPPH activity in all cultivars and varied significantly in pretreatments. Mabonde banana cultivar showed a low significant scavenging activity for citric acid pretreatment and at a concentration of 10 and 15 g/L. The DPPH scavenging activity also differed significantly (*P *<* *0.05) for Muomva-red and was highest (1.02 ± 0.01 mg GA/g with lactic acid concentration of 20 g/L). There was a significant difference in the antioxidant activities of cultivars as a result of organic acid pretreatment, with cultivars treated with lactic and ascorbic acids showing significantly higher antioxidant activity in all cultivars. IC_50_ is the amount of antioxidant necessary to decrease the initial DPPH absorbance by 50%. The lower the IC_50_ obtained during DPPH assay the better the scavenging property and ability to break the free radical chain reaction (Frankel 1991; Lim et al. 2007). The DPPH assay measures the ability of the extract to donate free hydrogen ions to the radical. During this assay, there is a reduction of the purple chromogen radical to pale yellow hydrazine by antioxidant compounds. This reduction activity of the purple chromogen radical corresponds to a decrease in optical density at long wavelengths (Musa et al. 2013).

**Table 4:** Effect of pretreatment concentration on DPPH^1^ scavenging activity of unripe noncommercial banana cultivars

Pretreatment concentration	Cultivars (mg GA/g d.w.)		
	Luvhele	Mabonde	Muomva-red
Ascorbic acid (g/L)			
10	0.81 ± 0.00^b^	0.17 ± 0.01^a^	0.87 ± 0.01^c^
15	0.66 ± 0.02^b^	0.55 ± 0.02^a^	0.85 ± 0.01^c^
20	0.84 ± 0.00^b^	0.81 ± 0.00^a^	0.95 ± 0.01^c^
Citric acid (g/L)			
10	0.79 ± 0.01^b^	0.16 ± 0.00^a^	0.92 ± 0.01^c^
15	0.97 ± 0.00^c^	0.12 ± 0.01^a^	0.92 ± 0.02^b^
20	0.90 ± 0.04^b^	0.89 ± 0.01^b^	0.50 ± 0.00^a^
Lactic acid (g/L)			
10	0.90 ± 0.00^b^	0.31 ± 0.01^a^	0.89 ± 0.02^b^
15	0.92 ± 0.00^b^	0.13 ± 0.00^a^	0.98 ± 0.01^c^
20	0.95 ± 0.01^a^	0.96 ± 0.02^a^	1.02 ± 0.01^b^

Means with same superscript across rows are not significantly different (*P *<* *0.05) using Tukey HSD test. Values are means ± standard deviation (*n* = 3).

1 DPPH - 1,1-diphenyl-2-picrylhydrazyl.

Generally, lactic acid pretreatment showed the highest DPPH activity (*P *<* *0.05) in all cultivars at pretreatment concentration of 20 g/L. DPPH scavenging activities of noncommercial cultivars were also significantly higher when compared to the results obtained by Moyo et al. (2013) on the antioxidant capacity of nonconventional leaves consumed in South Africa. The high DPPH scavenging activity of the noncommercial cultivars agrees with results obtained by Sarawong et al. (2014). Similar results obtained for DPPH scavenging activity were reported by Sulaiman et al. (2011) on eight Malaysian bananas using hexane, chloroform, and 80% methanol (v/v) as extraction solvent. The antioxidant capacity of banana cultivars as comparable to TPC is significantly affected by the mode of sample preparation as well as the solvent used for extraction. Accordingly, the distinctive properties of the structure and composition of individual plant materials make their behavior unpredictable when in combination with various solvents (González-Montelongo et al. 2010). Similarly, the overall antioxidant properties of food produce can be enhanced by the formation of products synthesized by Maillard reaction. Products formed from Maillard reaction are due mainly to intense heat treatment or prolonged storage, thus resulting in a strong antioxidant activity. Conversely, heat treatment and drying process can also result in a corresponding decrease of naturally occurring antioxidants (Nicoli et al. 1999; Toor and Savage 2006; Sulaiman et al. 2011).

## Conclusion

Significantly marked morphological differences exist among all the three noncommercial banana cultivars. Morphological profiles of different cultivars examined can be used as parameters for distinguishing these noncommercial banana varieties. The variations that exist in total polyphenol content (TPC) are other useful parameters needed for nutritional classification and differentiation. Among all cultivars examined, Muomva-red had the longest finger, highest amount of TPC, and the highest antioxidant activity. Compared to works done by other authors, high amount of total polyphenols (TPs) obtained in cultivars used in this study make them a suitable source of bio-nutrients with great medical and health function in the body. Recorded TPs in all samples were higher when compared to results obtained from other banana cultivars from different parts of the world. Thus, further processing, use and application of these noncommercial cultivars otherwise underutilized will be highly beneficial due to the presence of antioxidants.
